# Atom Probe Tomographic Mapping Directly Reveals the Atomic Distribution of Phosphorus in Resin Embedded Ferritin

**DOI:** 10.1038/srep22321

**Published:** 2016-02-29

**Authors:** Daniel E. Perea, Jia Liu, Jonah Bartrand, Quinten Dicken, S. Theva Thevuthasan, Nigel D. Browning, James E. Evans

**Affiliations:** 1 Environmental Molecular Sciences Laboratory, Pacific Northwest National Laboratory, Richland, WA 99352, USA; 2 Fundamental Computational Sciences Directorate, Pacific Northwest National Laboratory, Richland, WA 99352, USA

## Abstract

Here we report the atomic-scale analysis of biological interfaces within the ferritin protein using atom probe tomography that is facilitated by an advanced specimen preparation approach. Embedding ferritin in an organic polymer resin lacking nitrogen provided chemical contrast to visualise atomic distributions and distinguish the inorganic-organic interface of the ferrihydrite mineral core and protein shell, as well as the organic-organic interface between the ferritin protein shell and embedding resin. In addition, we definitively show the atomic-scale distribution of phosphorus as being at the surface of the ferrihydrite mineral with the distribution of sodium mapped within the protein shell environment with an enhanced distribution at the mineral/protein interface. The sample preparation method is robust and can be directly extended to further enhance the study of biological, organic and inorganic nanomaterials relevant to health, energy or the environment.

The ability to image biointerfaces over nanometer to micrometer length scales is fundamental to correlating biological composition and structure to physiological function, and is aided by a multimodal approach using advanced complementary microscopic and spectroscopic characterization techniques^1^,^2^,^3^,^4^. Atom probe tomography (APT) is a 3-dimensional compositional mapping technique based on the thermally-assisted field evaporation of individual atoms and/or compounds as positive ions from the tip of a cryogenically-cooled needle-shaped specimen^5^. The chemical identity of each ion is determined from time-of-flight mass spectrometry while the 3-dimensional position is determined from the relative position on the detector and the time sequence of evaporation events. From this information, a unique 3-dimensional reconstruction can be formed revealing the atomic scale composition along with impurity distributions up to a part-per-million sensitivity and subnanometer spatial resolution^6^,^7^. APT continues to make major contributions in the materials science of structural^8^,^9^ and electronic materials^8^,^10^, and recently has been demonstrated in geological^11^, bio-mineralogical^12^,^13^,^14^, soft organic^15^,^16^,^17^ and bio-organic^4^,^18^,^19^ materials. However the regular application of APT to soft biological materials is lacking in large part due to difficulties in specimen preparation.

The ferritin protein molecule has been the subject of previous Field Ion Microscopy (FIM) based analyses^20^,^21^ and more recent APT-based analyses^18^,^19^ because the iron-rich ferrihydrite core (hydrated iron (III) oxide) can provide an iron signature in the mass spectrum, the detection of which would confirm field evaporation of the protein. Additionally, through an accurate atom-by-atom reconstruction, the mineral core can also serve as a fiducial marker from which the relative composition of the organic protein shell can be tomographically mapped. The APT analysis of ferritin has the potential to directly reveal the subtle compositional distribution of phosphorus and other cations within and around the ferrihydrite mineral core and protein shell and thus provide insight into the presumed role of phosphorus in stabilizing the ferrihydrite structure^22^.

Here we report the atomic-scale tomographic analysis of biological materials using APT that is facilitated by a robust specimen preparation methodology involving the FIB-based preparation of needle-shaped specimens of horse spleen ferritin protein embedded in the organic polymer resin lowicryl K4M which lacks nitrogen in its structure. The premise behind this approach is that the tomographic distribution of iron from the protein provides chemical contrast to distinguish the inorganic-organic interface of the ferrihydrite mineral core and protein shell, while the organic-organic interface between the ferritin protein shell and the embedding resin is distinguished by tracking nitrogen content. In addition, we definitively show the atomic-scale distribution of phosphorus as being at the surface of the ferrihydrite mineral surface along with sodium being within the protein shell environment with an enhanced distribution at the mineral/protein interface. The results demonstrate a viable application of APT analysis to study the elemental distribution of complex biological interfaces involving organic macromolecules. We also show an extension of the specimen preparation technique can be applied to further enhance the study of non-biological organic and inorganic nanomaterials relevant to energy and the environment.

## Results

### Specimen preparation

**Figure 1:**
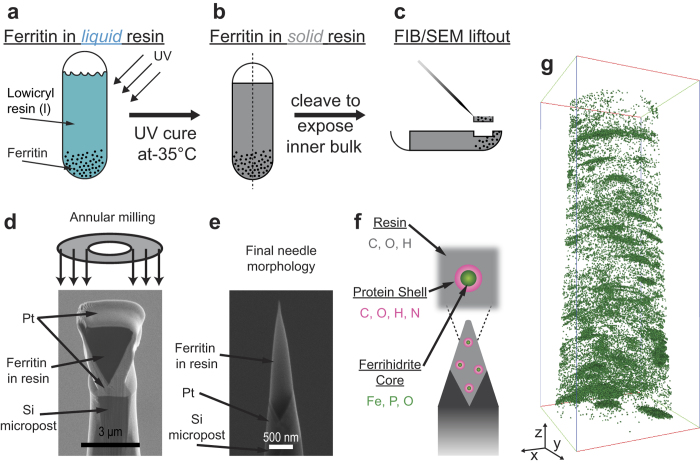
Preparation of specimens for APT analysis. (**a**) Ferritin is added to gelatin capsule followed by the addition of liquid lowicryl resin. (**b**) Specimen is cured at −35 °C under UV light exposure for 48 hours. (**c**) FIB/SEM is used to lift out a region of interest and (**d**) transfer onto individual Si microposts for annular milling to (**e**) create a needle shaped morphology with end radius of curvature ~100 nm. (**f**) Schematic representation of needle-shaped specimen containing a distribution of individual ferritin protein molecules. (**g**) 3D distribution of Fe determined by APT analysis. Bounding box dimensions are 49 × 48 × 134 nm^3^.

Specimens for APT analysis were fabricated using a dual beam focused ion/scanning electron microscope and consisted of individual ferritin protein molecules embedded in the lowicryl K4 M resin (Fig. 1). A detailed description of the ferritin molecule embedding process and specimen preparation procedure is given in the *Methods* section below. In brief, a concentrated ferritin mixture was combined with the lowicryl resin and UV cured at −35 °C (Fig. 1a) within a gelatin capsule. The specimen was then cleaved in half exposing the inside (Fig. 1b), where a FIB-based lift-out method was then used to liftout a lamellar wedge and fabricate individual needle-shaped specimens for APT analysis^23^ (Fig. 1c). Energy dispersive x-ray spectroscopy (EDX) was used to select a region-of-interest for lift out that showed a relatively high Fe signal coming from a high spatial concentration of ferritin cores. The use of XeF_2_ during large area trenching in the FIB was found to greatly enhance the success of prepared specimens, and is described in detail in the Methods section. Individual portions of the lifted out lamellar wedge were attached to individual micro-fabricated Si microposts, followed by annular milling to create a needle shaped morphology (Fig. 1d). An example of a typical specimen after annular milling is shown in Fig. 1e with an end diameter <100 nm. The final needle specimen composite consists of individual ferritin protein molecules embedded in the organic resin and is shown schematically in Fig. 1f. Specimens were also fabricated in parallel from pure lowicryl that *did not* contain the ferritin molecules (i.e. needles of resin without ferritin), as well as Fe_3_O_4_ nanoparticles embedded in the lowicryl resin to serve as controls for the APT analysis of ferritin. In an attempt to increase the mechanical stability and electrical conductivity, the needle specimens were conformally coated with ~10–20 nm of sputtered Cr metal prior to loading the specimens for APT analysis.

### APT mass spectral analysis of the pure resin

The goal of our work here is to use APT to detect and tomographically map the composition of biological interfaces, specifically ferritin molecules, with the ability to distinguish the bio organic macromolecules from the embedding organic polymer resin matrix. Our premise is to focus on the detection of specific key elemental signatures of iron and nitrogen–iron to spatially map the extent of the ferritin core, and nitrogen to distinguish the spatial extent of the protein shell from the embedding resin which *does not* contain any nitrogen. However, given the complex mass spectra that result from the pulsed-laser assisted field evaporation of organic polymer compounds using atom probe tomography^16^, the detection of multiple species having overlapping mass/charge ratio (m/z) can complicate or prevent a definitive identification of the nitrogen and iron species of interest. In addition, experimental parameters such as laser energy can also affect the observed distribution of mass peaks.

**Figure 2:**
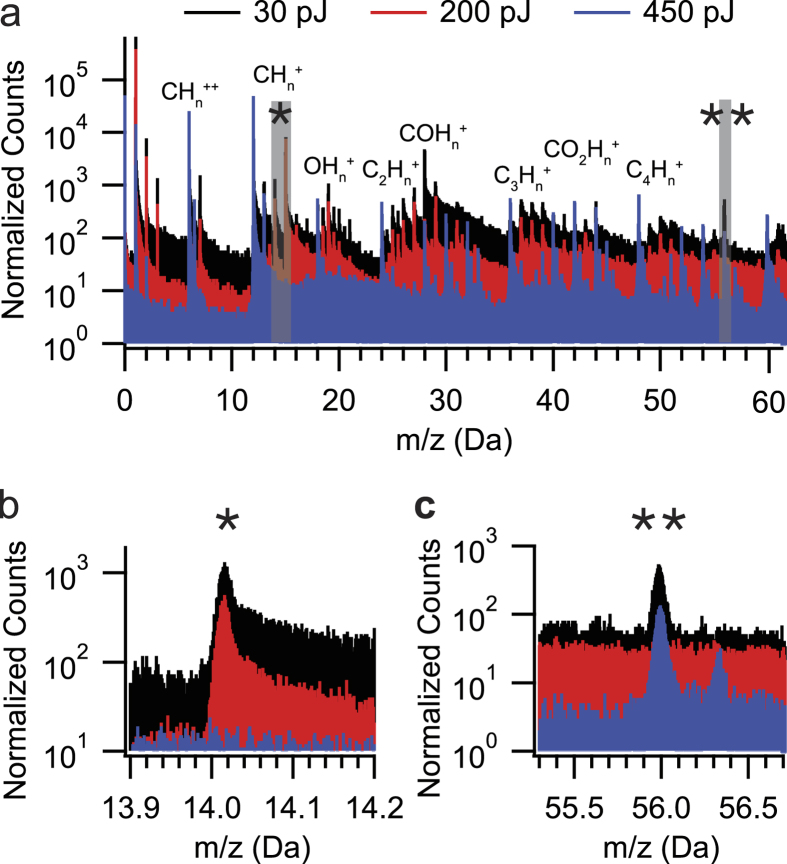
Comparing mass spectra from pure lowicryl at varying laser energy. (**a**) Mass spectra of lowicryl collected at 30 pJ (black), 200 pJ (red), and 450 pJ (blue) UV laser energies. Each spectrum is normalised to the same peak intensity of the 12C+ peak and contains approximately 10 M, 4 M, and 2 M. Total counts, respectively. A zoomed in region centered at (**b**) 14 Da and (**c**) 56 Da where 14N and 56Fe are expected, respectively. Since ferritin is not present in these pure lowicryl specimens, the observed peaks 14 and 56 Da are attributed to organic species.

**Figure 3:**
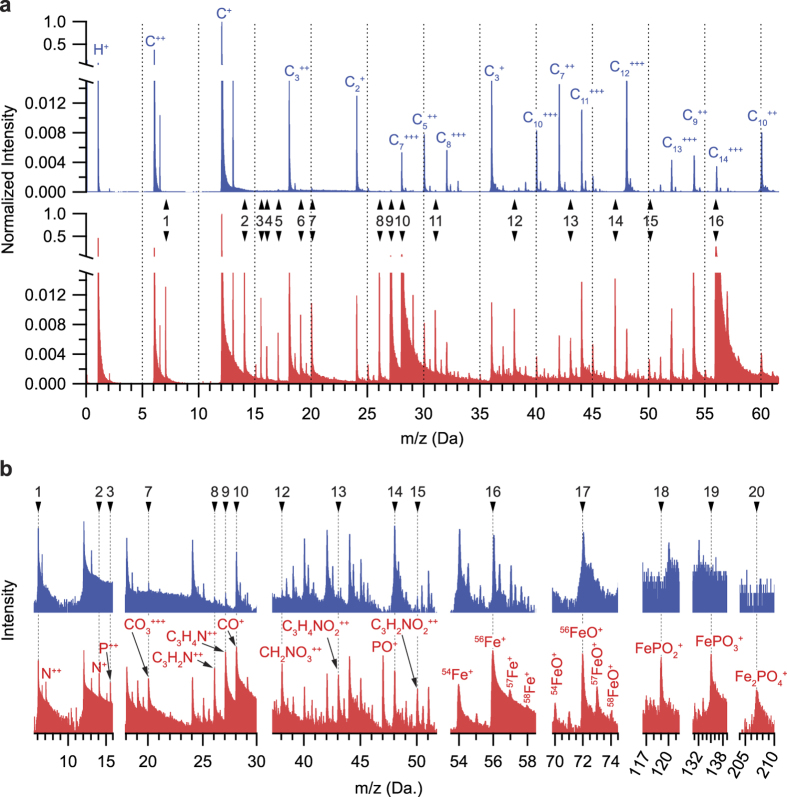
Comparing mass spectra from pure lowicryl and lowicryl embedded with ferritin. Mass spectra of (**a**) pure lowicryl (top; blue) and ferritin embedded in lowicryl (bottom; red) collected at 450 pJ UV laser energy. (**b**) zoomed in regions of peaks labeled in (**a**) for pure lowicryl (top; blue) and ferritin embedded in lowicryl (bottom; red). Potential peak identities of labeled peaks are listed in Supplementary Table 1.

In Fig. 2, the mass spectra of the pure embedding resin (i.e. without the ferritin) are shown for varying laser energies of 30 pJ, 200 pJ, and 450 pJ. Mass spectral peaks consistent with alkanes and carboxyl ions of varied saturations are observed, similar to a previous report of the APT analysis of poly(3-alkylthiophene)s reported by Prosa *et al*.^16^. However, at the higher energy of 450 pJ used here, we observed a clear decrease in the intensity of CH_n_ alkane peaks with n >1 compared at 30 pJ and 200 pJ (Fig. 2a), leading to C_n_ as the dominant signature (Fig. 3a; top curve). This result is in contrast to that reported by Prosa *et al*. where at increasing energy, no clear reduction or elimination in the saturated alkanes is observed. We note that while the laser energy seems to play a major role in the observed ionic fragmentation of the complex organic ions observed, the laser wavelength may also play an equally relevant role^24^ (532 nm for Prosa *et al*.; 355 nm used here); a detailed discussion of which is beyond the scope of the report here. Rather, we have focused on the observation that at 450 pJ, we are able to eliminate the mass spectral signature of CH_2_^+^ at 14 Da to maximise the ability of detecting any ^14^N^+^ signal resulting from the ferritin protein shell when analyzed. Figure 2b is zoomed in at the peak position expected for ^14^N^+^ (14.00 Da), where for both 30 pJ and 200 pJ laser energies, a peak associated with CH_2_^+^ (14.02 Da) is observed, while is absent in the 450 pJ mass spectra. In addition, a peak at 55.99 Da is observed in Fig. 2c to persist for all three laser energies, where ^56^Fe^+^ is expected. Since iron is not expected within the pure resin specimen, we associate this peak with C_14_^3+^ which is supported by the peak position at 56.33 Da being the ^12^C_13_^13^C^3+^ isotope. As we will show in the next subsection, the relatively small intensity C_14_^3+^ originating from the resin does not preclude the detection and spatial mapping of iron as ^56^Fe^+^ originating from the ferritin embedded resin.

The laser energy used in an APT analysis can have profound influence on the thermally-assisted field evaporation process which can be manifested as thermal artefacts in the mass spectrum such as thermal tails/humps or as artefacts in the tomographic reconstruction. The choice of 450 pJ laser energy was based on an empirical observation that a peak in the vicinity of 14 Da and 56 Da is minimised or eliminated as shown in Fig. 2b in the pure resin specimen, thus maximising the potential to detect a nitrogen signal from the protein shell of ferritin. No significant thermal artefacts such as large thermal humps or excessive thermal tails were observed in the full range mass spectra at 450 pJ shown in Supplementary Fig. 1a and b that could potentially complicate the identification of the mass peaks of interest.

### APT mass spectral analysis of the ferritin-embedded resin

At a laser energy of 450 pJ, C_n_ peaks of various charge states are observed as the dominant features from the pure resin specimen (Fig. 3a; top spectra). However when compared to the ferritin + resin specimen at 450 pJ (Fig. 3a; bottom spectra), many new peaks appear or show an increase in intensity which, by induction, result from both the ferritin protein shell and ferrihydrite mineral core. In Supplementary Figure 1c, the difference spectrum produced by subtracting the pure resin spectrum from the ferritin + resin spectrum in Fig. 3a is shown to qualitatively highlight the main peak differences and simulate a ferritin only spectra for visual comparison. Most of the new peaks that appear in the ferritin specimen are comprised of combinations of C, N, O, and H, consistent with the amino acid building blocks which make up the 24 peptide subunits of the protein shell. These dominant new peaks positions are highlighted by the numbered arrows and the possible peak identities are labeled in Fig. 3b and summarised in Supplementary Table 1.

Notably, in Fig. 3b, distinct N^+^ (14.00 Da) and N^++^ (7.00 Da) ions are observed in the ferritin specimen and are noticeably absent from the pure resin. A strong Fe signal comprised of the four expected isotopes is also observed, providing definitive indication that the ferritin core was detected. Inorganic phosphorus is also detected as FePO_2_^+^, FePO_3_^+^, and Fe_2_PO_4_^+^, as well as PO^+^, and elemental P^++^ ions. It is clear that mass spectral signatures consistent with the ferritin protein are observed relative to the spectrum of the pure resin specimen. In the section that follows, we focus on the atomic-level spatial distribution of these signatures to reveal the compositional profile of the ferritin protein, including a subtle but distinct distribution of N delineating the interface between the bio-organic protein shell and the organic polymer resin.

### 3D distribution of Fe and radial compositional distribution of ferritin

The 3D atom probe tomographic reconstruction reveals regions enriched with Fe, consistent with the iron-rich ferrihydrite mineral core of the ferritin molecules (Fig. 1g). To perform a composition profile analysis, a 15% ^56^Fe^+^ isoconcentration surface was used to enclose individual Fe-rich regions. Proximity histogram (proxygram) analysis^25^ was then applied to create a 1D compositional profile from bins of a specified width and step distance following the morphological contours defined by the vector normal of the isoconcentration polyhedra. The isoconcentration surfaces for the whole reconstruction as well as an isolated surface are shown in Supplementary Fig. 2.

**Figure 4:**
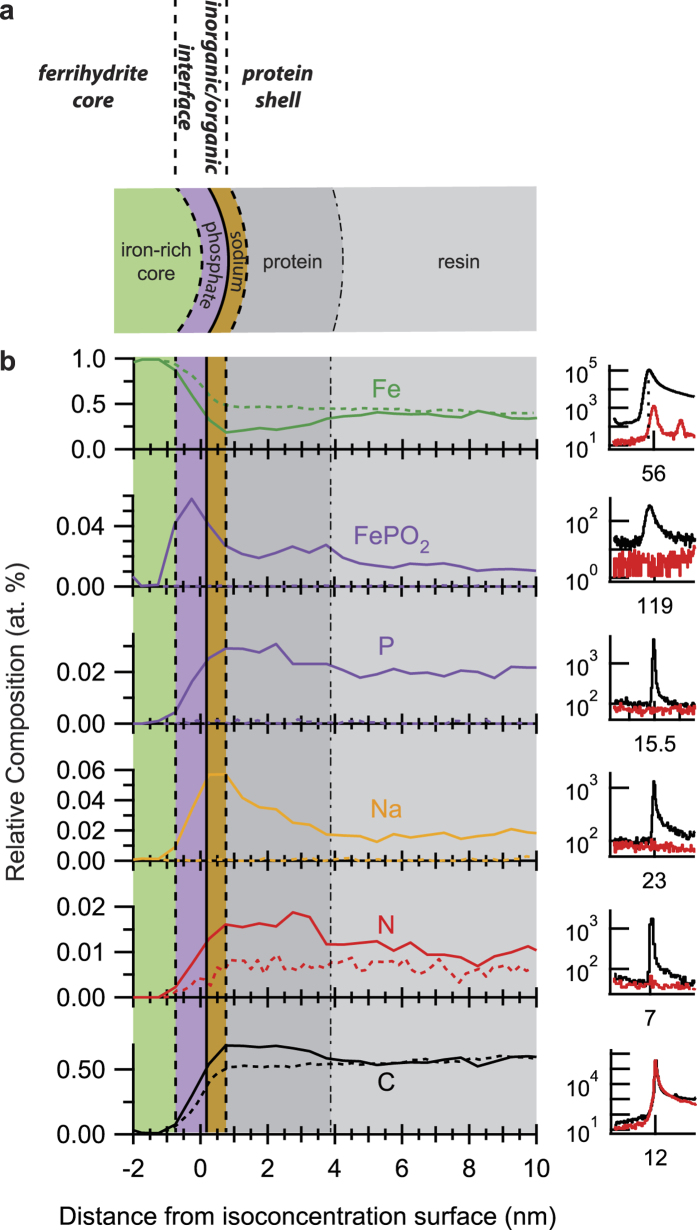
Composition profile of the ferritin protein from APT analysis. (**a**) Schematic illustration outlining compositionally-distinct radial regions of the ferritin protein. (**b**) Proxygram composition profiles for Fe, FePO_2_, P, Na, N, and C from the ferritin embedded in lowicryl specimens. Corresponding regions of the mass spectra are shown to the right of each profile for the resin embedded with ferritin (black) and the pure lowicryl (red). The composition profiles taken from the Fe_3_O_4_ specimen are shown as the dashed curves.

Proxygram analysis of the ferritin-embedded resin specimens reveals the compositional profile of the ferritin/resin system schematically illustrated in Fig. 4a. In Fig. 4b, the average of proxygram profiles from 10 individual isoconcentration surfaces from the ferritin embedded in resin sample are shown for Fe, FePO_2_, P, Na, N, and C (solid lines). Note that isoconcentration surfaces that are truncated by intersection with the edge of the 3D reconstruction and do not result in a fully enclosed isoconcentration surface are excluded from proxygram analysis. The dashed curves are profiles produced from Fe_3_O_4_ nanoparticles embedded in resin, and are described below. Corresponding portions of the mass spectra are shown on the right for each composition profile to compare the mass spectra of the pure resin specimen (red) to the ferritin embedded in the resin (black). For FePO_2_, P, Na, N, and C, there is a clear associated peak in the ferritin embedded in resin sample, the maximum of which is at least an order of magnitude greater than the pure resin specimen, and supports and unambiguous compositional profile originating from the ferritin. Note that there is a slight, but measureable offset in the peak position of the ^56^Fe^+^ peak of the ferritin in resin sample at 55.94 Da, compared to the C_14_^+++^ peak at 56.00 Da, highlighted by the vertical dashed line. Since we only focus on specific peak signals which show an unambiguous difference between the ferritin and pure resin specimens, the profiles are reported as relative composition and do not represent the actual composition. The position at 0 nm represents the position of the defined 15% ^56^Fe^+^ isoconcentration surface, with negative (positive) values spanning inside (outside) the surface. Between −2 nm to about −1 nm, the composition is dominated by Fe indicative of the ferrihydrite mineral core (green fill). Between −1 nm to about 1 nm, the Fe intensity decreases while the rest of the species show an increase, marking the inorganic/organic interfacial region between the ferrihydrite mineral core and the protein shell. Beyond the position of 1 nm, the Fe intensity increases over about 4 nm before it plateaus and likely results from averaging multiple individual ferritin molecules with variable ferritin-to-ferritin spacing to generate the proxygram in Fig. 4b.

The surface of the ferrihydrite mineral core is enriched in P (purple fill) followed by Na (orange fill), forming a transition region between the ferrihydrite mineral core and the protein shell. The P-containing mass spectral peaks of FePO_2_, P are used to delineate the P profile. The FePO_2_ profile shows a maximum just before 0 nm followed by a rise of the P profile. The P-containing profiles of the horse spleen ferritin are strongly suggestive of a P enrichment at the ferrihydrite mineral surface, and is consistent with previous *indirect* determinations of P enriched surfaces of mammalian ferritins^26^,^27^, as opposed to P distributions that penetrate throughout the ferrihydrite core in bacterioferritins^27^. The relatively sharp peak of the FePO_2_ profile is suggestive as P being bound as iron phosphate at the surface. The P-rich surface is followed by a peak in the Na profile highlighted by the orange filled region in Fig. 4. To the best of our knowledge, the presence and distribution of Na within horse spleen ferritin has not been previously reported, but may not be too surprising considering the role Na plays in the ionic transport^28^ and biomineralization process^29^. We note the possibility that Na may be an artefact introduced from the specimen preparation process. The presence of Na has been shown in the specific formulation of Lowicryl HM20 using X-ray microanalysis^30^. However in our case, we are using MonoStep Lowicryl K4M, which has a different formulation from HM20 and does not show the presence of Na within the APT spectra of the pure resin as indicated by the lack of a mass peak (see black mass spectra to the right of the Na proxygram composition profile of Fig. 4b). We also note that Na could possibly be introduced through buffer solutions used during the experiments or other contaminating sources. However, if Na is being incorporated from the buffer into the specimen, one may expect to see either a constant value of Na enrichment from the inorganic core to the external buffer or a compositional gradient that increases continuously from the inorganic core to the external buffer. In our case, we see the opposite since a clear spike in composition only occurs at the surface of the ferrihydrite mineral core which is suggestive that the presence of Na is specific. In addition, Gordon *et al*.^12^ have recently reported the co-localization of Na with Fe from magnetite and C from nanometer-sized organic fibers in the tooth of a marine mollusk. We have repeated the APT analysis of ferritin embedded in lowicryl, where again we observe the localization of Fe consistent with the iron-rich ferrihydrite core (Supplementary Fig. 4a), along with similar composition profiles (Supplementary Fig. 4b,c). Therefore, while we cannot discount the possibility that the Na distribution measured here in ferritin is physiological, additional experiments are needed to confirm the full mechanism of Na sequestration and its physiological impact.

The extent of the organic protein shell is delineated by the N profile in Fig. 4b and is highlighted by the dark grey fill. Between approximately 1 nm and 4 nm, the N profile shows a plateau, and then decreases after a position of 4 nm. The C profile shows a similar trend, although more subtle decrease after 4 nm due to the relatively large background signal coming from the organic embedding resin, evidenced by the mass spectra to the right of the C profile. To further support these results, we repeated the same proxygram analysis of the Fe_3_O_4_ nanoparticles embedded in lowicryl, the reconstructed volume and ^56^Fe^+^ isoconcentration surfaces of which are shown in Supplementary Fig. 3. The proxygram compositional profiles averaged from 51 individual nanoparticles are shown as the dashed curves in Fig. 4b, with only the Fe, N, and C profiles from the Fe_3_O_4_ particles showing a significant signal. Interestingly, the Fe_3_O_4_ particles do show a signal from N (7 Da) which we have confirmed from the manufacturer, that N is present in the organic ligands surrounding the nanoparticles. However, in this case, the N profile from the Fe_3_O_4_ sample is constant. A comparison of the N and C profiles between the Fe_3_O_4_ (dashed) and ferritin (solid) sample support the delineation of the compositional extent of the protein shell observed for the ferritin.

## Discussion

In summary, we have reported a significant advancement in the atomic-scale tomographic analysis of biological materials using atom probe tomography (APT) that is facilitated by an advanced specimen preparation approach. A novel specimen preparation strategy is used in the APT analysis of horse spleen ferritin protein embedded in an organic polymer resin. We show that the ferritin protein molecules can be field evaporated and tomographically reconstructed in a local electrode atom probe. The distribution of Fe provided an unambiguous fiducial signature of the ferritin ferrihydrite core, allowing us to map the compositional profile of both the inorganic-organic interface of the ferrihydrite mineral core and protein shell, as well as the organic-organic interface between the ferritin protein shell and embedding resin. In addition, we *directly* map the distribution of phosphorus as being at the surface of the ferrihydrite mineral. We also report the distribution of sodium within the protein shell environment with an enhanced distribution at the mineral/protein interface.

The results demonstrate a viable application of APT analysis to study complex biological interfaces, including organic-organic interfaces at the atomic level and part-per-million sensitivity. An extension of the specimen preparation technique reported here can further enhance the APT study of organic and inorganic materials and nanoparticles relevant to health, energy and the environment. We envision that the specimen preparation technique described here can be adapted to larger, more complex biological systems using isotopic labeling to generate the chemical contrast. Furthermore, until now, the APT analysis of freestanding nanoparticles has remained challenging and is often achieved only via the serial manipulation of individual nanoparticles which is time consuming or by embedding ensembles of nanoparticles between metal layers which can cause potential artifacts or low analysis yields. However, the method shown here mitigated both of these issues during the analysis of dispersed Fe_3_O_4_ nanoparticles thereby heralding a new route for freestanding nanoscale specimen preparation for APT analysis which will likely have broad impact toward accelerating the study of organic and inorganic nanomaterials and catalysts with APT. In addition, the ability to map atomic-level compositional profiles provides an analytical approach to directly quantify chemical gradients in biological systems which underlie biological function at a cellular level, the analysis of which may be enhanced through isotopically-labeling and/or cryo-based embedding and sample preparation strategies.

## Methods

### Polymerization of pure lowicryl

MonoStep Lowicryl K4M used in this study was purchased from Electron Microscopy Sciences (14335) and requires no preparation or mixing. Using a micropipette, 100 μL of lowicryl was injected into a gelatin capsule and immediately purged with dry N_2_ gas and capped. The specimen was then placed within a Leica EMAFS freeze substitution system. The lowicryl resin was polymerised by direct exposure to long-wave length UV-irradiation (10 watt) at −35 °C for 48 hours. The resulting hardened lowicryl was then cut in half along the length of the capsule using a razor blade to expose the inside core and ~10 nm of sputter coated carbon was deposited to mitigate electrical charging during imaging in the dual beam FIB/SEM. The specimen was then loaded into a dual beam FIB/SEM for site-specific liftout and preparation of APT specimens as described below.

### Polymerization of ferritin embedded in lowicryl

Cationised ferritin from horse spleen was used in this study and purchased from Sigma Aldrich; 10 mg/mL in 0.15 M NaCl (F7879-2ML). To embed the ferritin into the lowicryl, 200 μL of ferritin solution was centrifuged for 30 min in a mini centrifuge at (Fisher Scientific) until the ferritin molecules were visually concentrated at the bottom of centrifuge vial. The clear supernatant was removed and replaced with 10 μL of isopropanol and mixed to re-suspend. 2 μL of mixed solution was then transferred via pipette to a clean gelatin capsule, followed by the addition of 98 μL of lowicryl. Care was taken to not completely mix the lowicryl with the ferritin, so that a high concentration of ferritin remains at the bottom of the capsule. The sample was then UV-cured and prepared for imaging following the same procedure as for the pure lowicryl.

### Polymerization of Fe_3_O_4_ nanoparticles embedded in lowicryl

Fe_3_O_4_ nanoparticles of 10 nm diameter were used in this study and purchased from Nanomaterials & Nanofabrication Laboratories (FEOW-10-025). 100 μL of Fe_3_O_4_ suspended was mixed with 100 μL of isopropanol and centrifuged for 30 min leaving a concentrated solution at the bottom of the centrifuge vial. The clear supernatant solution was removed and replaced with 10 μL of isopropanol to make a uniform solution. 2 μL of mixed solution was then transferred via pipette to a clean gelatin capsule, followed by the addition of 98 μL of lowicryl. Care was taken to not completely mix the lowicryl with the nanoparticles, so that a high concentration of nanoparticles remains at the bottom of the capsule. The sample was then UV-cured and prepared for imaging following the same procedure as for the pure lowicryl.

### FIB/SEM preparation of specimens for APT analysis

Prior to FIB/SEM imaging, the polymerised lowicryl was cut in half along the length of the capsule using a razor blade to expose the inside core followed by the sputter coating of ~10–20 nm of carbon to mitigate electrical charging during imaging. The specimen was then loaded into a FEI Quanta dual-beam focused ion beam/scanning electron microscope equipped with an OmniProbe 200 manipulator, EDX analysis system, and a Pt and XeF_2_ gas injection source.

For the embedded ferritin and Fe_3_O_4_ nanoparticle samples, regions of interest for specimen liftout were identified using Energy Dispersive X-Ray analysis (EDX) to map the relative intensity of Fe using the Fe-L edge. In this way, regions were targeted with high Fe content to increase the probability of capturing the ferritin and nanoparticles within the analyzed APT volume. Electron-beam-assisted deposition (EBAD) of Pt/C from the gas injection source gas (C_5_H_4_)CH_3_Pt(CH_3_)_3_ was used to create a protective capping layer of approximately 3 × 20 μm^2^ and 200 nm thick over a region of interest.

FIB-based specimen preparation of lowicryl-based specimens for APT analysis was performed following a procedure described by Thompson *et al*.^23^, but was modified with the use of XeF_2_ to enhance preparation of the polymer-based specimens. The relatively soft and electrically insulating nature of the lowicryl resin makes it challenging to mill the necessary trenches for liftout effectively, resulting in non-uniform milling rates resulting in the formation of pits and voids. The generalised FIB-based approach to prepare specimens from polymers can lead to sample overheating which results in morphological instabilities, where alternative mill patterning approaches to minimise ^69^Ga^+^ ion exposure can be used as a mitigation strategy^31^. An alternative approach is to use a gas-assisted etching (GAE) approach using XeF_2_ to enhance the milling of polymer materials^32^. We found that exposure of XeF_2_ during the milling process improved the milling rate by a factor of 3–5 times and improved the morphological uniformity of the milled area, allowing successful fabrication of needle-shaped specimens with tip diameters <100 nm for APT analysis.

Trenching of the lowicryl to create a lamellar wedge was performed with a 8 kV ^69^Ga^+^ ion beam and currents between 0.27–0.75 nA while the whole time exposed to XeF_2_ gas. Portions of the lamellar wedge were attached to individual microposts on specialised substrates purchased from Cameca Instruments, Atom Probe Tomography Division (part#: 23264). Each micropost with a sample was then annular milled using an 8 kV ^69^Ga^+^ ion beam, followed by a 2 kV exposure to remove the remaining Pt protective cap. In an attempt to increase the mechanical stability and electrical conductivity, a conformal 10–20 nm Cr coating was sputtered on sharpened tips using an Ion Beam Sputtering/etching (IBS/e) system from South Bay Technologies prior to loading specimens for APT analysis.

### Atom Probe Tomographic Analysis and Data Reconstruction

APT analysis of lowicryl-based specimens was performed using a LEAP 4000X-HR equipped with a pico-second 355 nm UV laser from Cameca Instruments^33^. Vacuum pressure in the analysis chamber was <2 × 10^−11^ Torr. The specimen was cooled to a set point of 40 K (actual 44.1 K). Laser energy ranged between 0.2–400 pJ at a frequency of 160 kHz and a detection rate of 320–480 ions/sec (0.2–0.3%). The analysis resulted in 3–5 M detected ions. Specimens were analyzed to the point of specimen fracture, terminating the analysis.

The Integrated Visualization and Analysis Software (IVAS) from Cameca Instruments were used to reconstruct the data. A semi-quantitative reconstruction scaling was performed using the *tip profile* method to determine the reconstructed radius as a function of analyzed depth^34^.

## Additional Information

**How to cite this article**: Perea, D. E. *et al*. Atom Probe Tomographic Mapping Directly Reveals the Atomic Distribution of Phosphorus in Resin Embedded Ferritin. *Sci. Rep.* **6**, 22321; doi: 10.1038/srep22321 (2016).

## Supplementary Material

Supplementary Information
