# Supplementary material for: Atom Probe Tomographic Mapping Directly Reveals the Atomic Distribution of Phosphorus in Resin Embedded Ferritin
Source: Sci Rep. 2016 Feb 29;6:22321. doi: 10.1038/srep22321 (PMC4770421; doi:10.1038/srep22321)
Supplement: Supplementary Information [file srep22321-s1.pdf]

Supplementary Information

Atom Probe Tomographic Mapping Directly Reveals the Atomic Distribution of Phosphorus in Resin Embedded Ferritin

Daniel E. Perea[1,*], Jia Liu[1], Jonah Bartrand[1], Quinten Dicken[1], S. Theva Thevuthasan[1,#], Nigel D. Browning[2] and James E. Evans[1]*

[1] Environmental Molecular Sciences Laboratory, Pacific Northwest National Laboratory, Richland, WA 99352, USA

[2] Fundamental Computational Sciences Directorate, Pacific Northwest National Laboratory, Richland, WA 99352, USA

[#] Current Address: Qatar Environment and Energy Research Institute, Qatar Foundation, Doha, Qatar

* Corresponding Authors: D.E.P.: daniel.perea@pnnl.gov; J.E.E.: james.evans@pnnl.gov

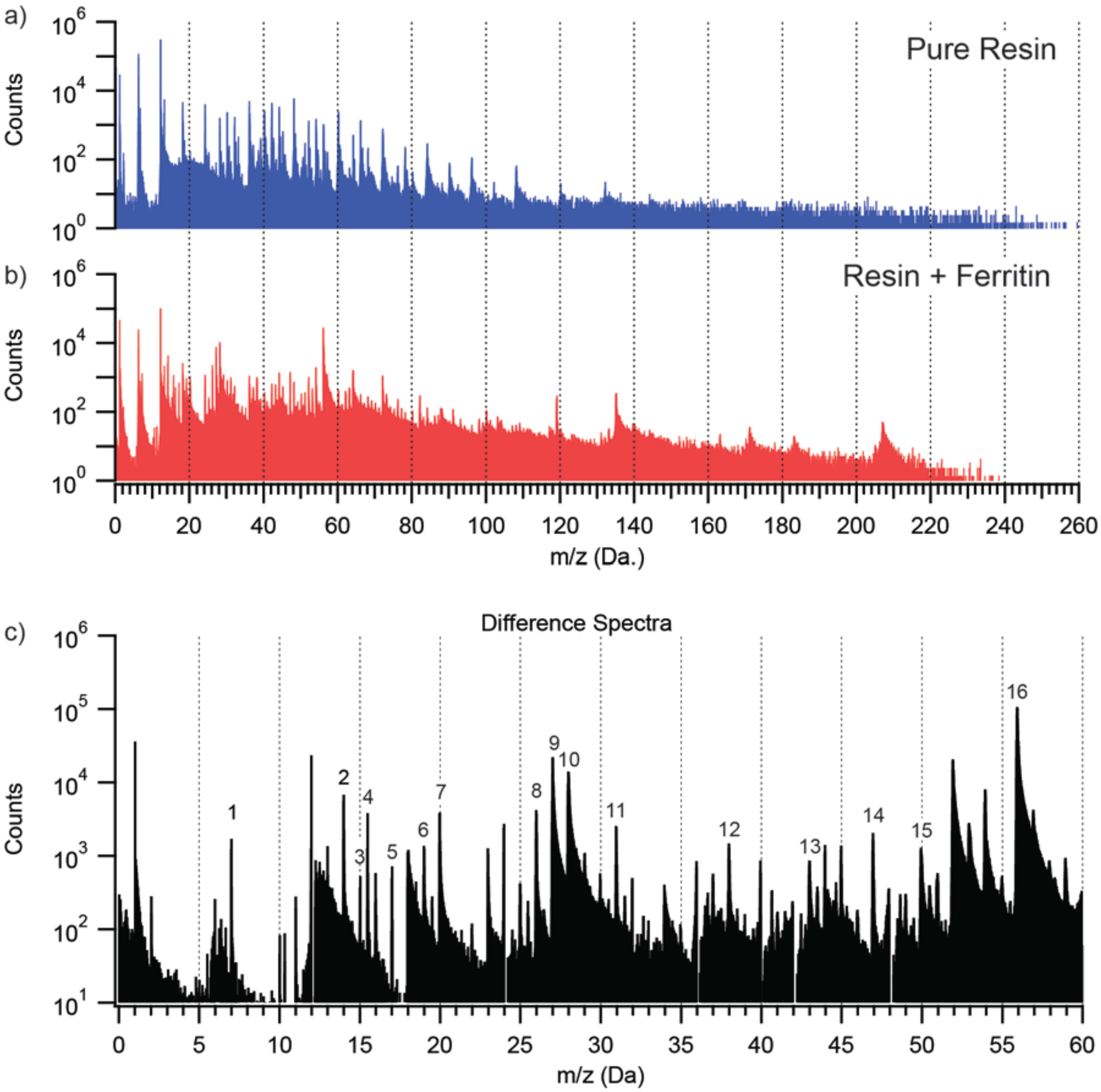

Supplementary Figure 1 | Comparing mass spectra from pure resin and ferritin embedded resin. Mass spectra showing the full detected mass range for the a) pure resin and b) the ferritin embedded in resin specimens collected using 450 pJ UV laser energy. The spectra show the full mass range of data collected and are the same as those shown in Figure 3a of the main text. c) Difference spectra produced by subtracting the mass spectrum in a) from the mass spectrum in b). The numbered peaks correspond to the same peaks in Figure 3 of the main text.

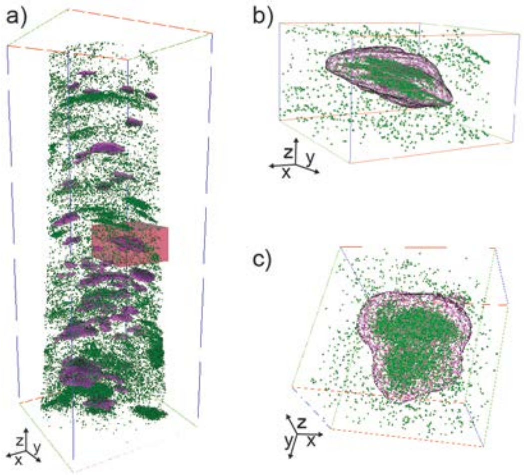

Supplementary Figure 2 | Tomographic distribution of Fe in ferritin specimen. a) Tomographic reconstruction showing the distribution of individual Fe atoms (green) from ferritin embedded in lowicryl. Bounding box dimensions are 49×48×134 nm^3. 15% $^{56}Fe^+$ isoconcentration surfaces composed of many polyhedra enclose regions rich in Fe. Isoconcentration surfaces that are truncated by intersection with the edge of the 3D reconstruction and do not result in a fully enclosed isoconcentration surface are excluded from proxygram analysis. **b)** and **c)** show different orthogonal views of the isolated 15% Fe isoconcentration polyhedra highlighted by the shaded cube in a). Bounding box dimensions 20×20×12 nm^3.

15% $^{56}Fe^+$ isoconcentration polyhedra (purple mesh) are used to enclose and outline the morphological contours of the Fe rich regions and are shown in Supplementary Fig. 2a. The Fe-rich regions do not have a spherical-like morphology as may be expected; rather they are reconstructed to have a more ellipsoidal-like shape with the elongated face oriented normal to the analysis direction. This is better illustrated in Supplementary Fig. 2b and 2c of an isolated Fe core shown from two different orthogonal perspectives. Analysis of Fe_3O_4 nanoparticles embedded in lowicryl also exhibit a similar reconstructed morphology (Supplementary Fig. 3). The ellipsoidal-like shape of both the ferritin core and the magnetite nanoparticles is likely an aberration of the reconstruction resulting from evaporation bursts that occurred during the field evaporation of the relatively-high field Fe cores embedded in the relatively low field organic polymer. We note that Green et al also reported similar evaporation bursts during the field evaporation of ferritin embedded in solid NaCl[19]. Despite any likely reconstruction artifacts, the evaporation bursts do not seem to strongly perturb the measured spatial distributions of key elements, the composition profiles of which are averaged over many individual ferritin molecules.

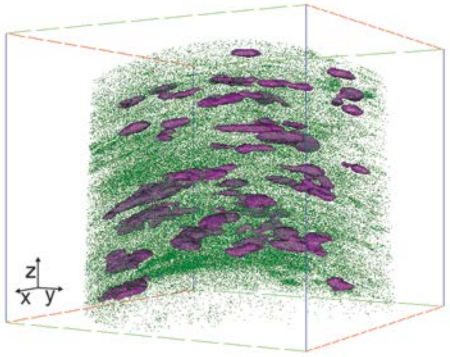

Supplementary Figure 3 | APT reconstruction of individual Fe_3O_4 nanoparticles. Tomographic reconstruction showing the distribution of individual Fe atoms (green) from Fe_3O_4 nanoparticles embedded within lowicryl. Reconstruction bounding box dimensions are 66×65×56 nm^3. 15% $^{56}Fe^+$ isoconcentration surfaces enclose regions rich in Fe (purple).

To compare with the morphology of the reconstructed Fe core from ferritin, we prepared and analyzed specimens consisting of inorganic Fe_3O_4 nanoparticles embedded in lowicryl. Supplementary Fig. 3b shows a reconstructed ion map of Fe from 10 nm diameter Fe_3O_4 nanoparticles embedded in lowicryl, which exhibit a qualitatively similar disc-like morphology compared to the Fe distribution measured from the ferritin specimens shown in Supplementary Fig. 2.

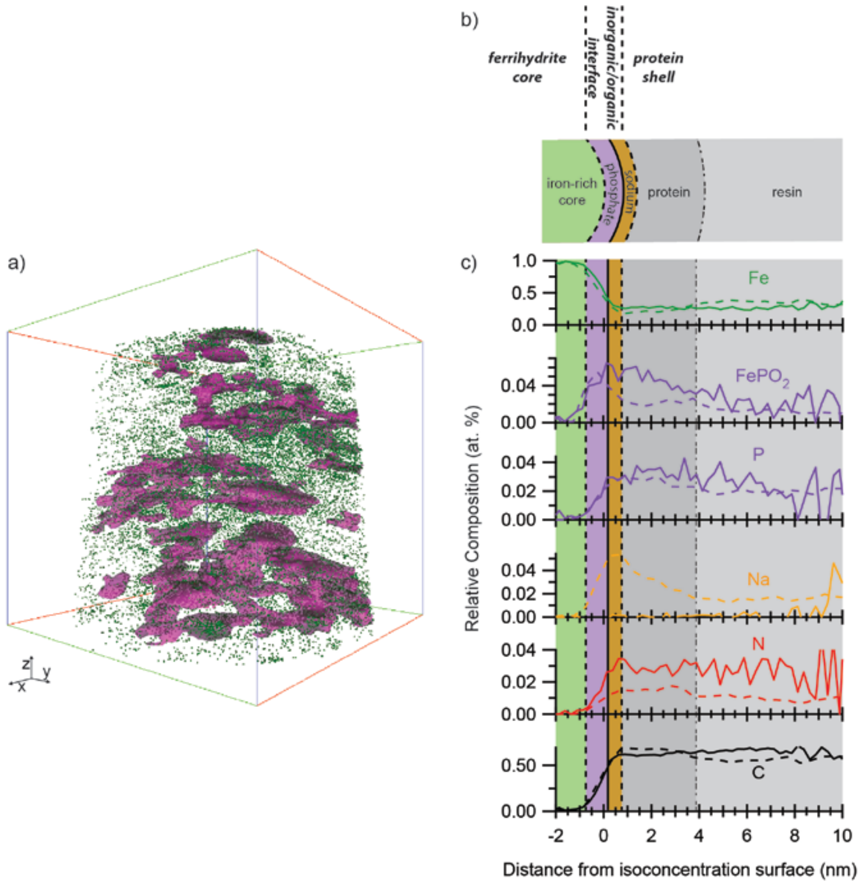

Supplementary Figure 4 | Repeatability of APT analysis of ferritin embedded in lowicryl resin. a) Tomographic reconstruction showing the distribution of individual Fe atoms (green) from ferritin and 15% Fe isoconcentration surfaces are used for proxygram analysis. The APT analysis here is from a different specimen as the one presented in Fig. 4 of the main paper. Reconstruction bounding box dimensions are 48×48×46 nm^3. **b)** Schematic illustration outlining compositionally-distinct radial regions of the ferritin protein. **c)** Proxygram composition profiles for Fe, $FePO_2$, P, Na, N, and C from the ferritin embedded in lowicryl specimens shown in a) here (solid curves) and from the Fig 4b (dashed curves)

In Supplementary Fig. 4a, the 3D compositional map of Fe is shown from a specimen tip that is different from the analysis presented in the main text, but prepared from the same bulk liftout. One-to-one comparisons of the composition profiles between the two specimens are shown in Supplementary Fig. 4c, with the solid-line profiles from the repeated analysis, and the dashed-line profiles from Fig. 4b of the main text. The $FePO_2$, P, and C composition profiles are spatially consistent between the two specimens even though the relative ferritin-to-ferritin spacing is significantly different between the 2 datasets. Interestingly, the N and Na composition profiles appear to be partially obscured due to the higher particle density with variable interparticle spacings. In particular, the Na peak shows a near constant background level from -2 to +8 nm on the isoconcentration surface then shows a dramatic peak from +8 to +10 nm. This region would correspond to the bulk resin and there is no physical reason why Na would sequester in a shell ~5nm beyond the ferritin protein shell. It is likely therefore that the denser particle packing in the current reconstruction is obscuring the interpretation. A similar effect may

be occurring with the N peak which shows a similar rise from the core to the protein shell as seen in Figure 4b, but does not drop to a background level beyond the protein-resin interface even though the resin itself has been shown to lack any N.

Supplementary Table 1 | Potential spectral mass peak identities for the peaks labeled in Fig 3.

Peak label	m/z	Potential ion identity
**1**	7.00	N^{2+}, CH_2^{2+}
**2**	14.00	N^{+}, CO^{2+}, CNH_2^{2+}, CNO^{3+}, $CN_2H_2^{3+}$, $C_2OH_2^{3+}$
**3**	15.49	P^{2+}
**4**	15.99	O^{+}, PH^{2+}, POH^{3+}, $NO_2H_2^{3+}$, NPH_3^{3+}, C_4^{3+}
**5**	17.00	OH^{+}, PH_3^{2+}, CNH^{3+}, $C_4H_3^{3+}$ POH_4^{3+}
**6**	19.01	OH_3^{+} ,$C_2N_2^{+}$, $C_3H_2^{2+}$, CN_2OH^{3+} ,$C_2NOH_3^{3+}$, $C_2N_2H_5^{3+}$, $C_3OH_5^{3+}$
**7**	19.99	C_2O^{2+}, $N_2O_2^{2+}$, CO_3^{3+}, C_5^{3+}, PN_2H^{3+}, $COPH^{3+}$
**8**	26.00	CN^{+}, C_3O^{2+}, $C_3H_2N^{2+}$, PNO_2H^{3+}, $N_2O_3H_2^{3+}$, $PON_2H_3^{3+}$, $PO_2CH_3^{3+}$,
**9**	27.01	CNH^{+}, CN_3^{2+}, $C_3OH_2^{2+}$, $C_3NH_4^{2+}$, $C_2N_4H^{3+}$, $C_3N_2OH^{3+}$, $NO_4H_3^{3+}$, $C_4NOH_3^{3+}$, $N_2O_3H_5^{3+}$, $C_4N_2H_5^{3+}$, $C_5OH_5^{3+}$
**10**	28.00	Fe^{2+}, N_2^{+}, CO^{+}, CN_2O^{2+}, C_2PH^{2+}, $C_2NOH_2^{2+}$, CN_4O^{3+}, $C_2N_2PH^{3+}$, C_3POH^{3+}, $C_2N_3OH_2^{3+}$, $C_3NO_2H_2^{3+}$, $C_3NPH_3^{3+}$, $C_4O_2H_4^{3+}$, $PO_3H_5^{3+}$, $C_4PH_5^{3+}$
**11**	30.97	P^{+}
**12**	38.00	C_2N^{+}, $N_2O_3^{2+}$, $C_4N_2^{2+}$, C_5O^{2+}, PN_2OH^{2+}, $N_3O_2H_2^{2+}$, $CNO_3H_2^{2+}$, $C_5NH_2^{2+}$, $PN_3H_3^{2+}$, $CNPOH_3^{2+}$, $C_2O_3H_4^{2+}$, $C_2POH_5^{2+}$, $C_2N_3O_3^{3+}$, $C_3NO_4^{3+}$, $C_2N_3POH^{3+}$, $C_3NPO_2H^{3+}$, $C_2N_4O_2H_2^{3+}$, $C_3N_2O_3H_2^{3+}$, $C_4O_4H_2^{3+}$, $C_2N_4PH_3^{3+}$, $C_3N_2POH_3^{3+}$, $C_4PO_2H_3^{3+}$
**13**	43.01	N_3H^{+}, $CNOH^{+}$, $C_2OH_3^{+}$, $CN_4OH_2^{2+}$, $C_3O_3H_2^{2+}$, $C_2N_2PH_3^{2+}$, $C_2N_3OH_4^{2+}$, $C_3NO_2H_4^{2+}$, $C_3NPH_5^{2+}$, $C_4PH_7^{2+}$, $C_2N_4O_3H^{3+}$, $C_4NO_4H_3^{3+}$, $C_3N_3POH_4^{3+}$, $C_3N_4O_2H_5^{3+}$, $C_4N_2O_3H_5^{3+}$, $C_5O_4H_5^{3+}$, $C_3N_4P_2H_6^{3+}$, $C_4N_2OPH_6^{3+}$, $C_5O_2PH_6^{3+}$, $C_4N_3O_2H_7^{3+}$, $C_5P_2H_7'^{3+}$, $C_5NO_3H_7^{3+}$, $C_4N_3PH_8^{3+}$, $C_5NOPH_8^{3+}$
**14**	46.97	PO^{+}, P_3H^{2+}, $C_4P_3^{3+}$, P_4OH^{3+}, $P_2NO_4H^{3+}$
**15**	50.01	C_3N^{+}, $O_3H_2^{+}$, $C_4H_2^{+}$, NPH_5^{+}, $CN_4O_2^{2+}$, $C_2N_3O_2H_2^{2+}$, $C_3NO_3H_2^{2+}$, $C_2N_3PH_3^{2+}$, $C_2N_4OH_4^{2+}$, $C_3N_2O_2H_4^{2+}$, $C_4O_3H_4^{2+}$, $C_3N_2PH_5^{2+}$, $C_4POH_5^{2+}$, $C_4NO_2H_6^{2+}$, $C_4NPH_7^{2+}$, $C_5N_4O_2H_2^{3+}$, $C_5N_4PH_3^{3+}$, $C_2N_3PO_3H_5^{3+}$, $C_2N_4O_4H_6^{3+}$, $C_3N_2PO_3H_7^{3+}$, $C_4O_4PH_7^{3+}$, $C_3N_2P_2OH_8^{3+}$, $C_3N_3O_4H_8^{3+}$, $C_4O_2P_2H_8^{3+}$, $C_4NPO_3H_9^{3+}$
**16**	55.93	Fe^{+}
**17**	71.94	FeO^{+}
**18**	118.90	$FePO_2^{+}$, FeP_2H^{1+}
**19**	134.90	$FePO_3^{+}$, FeP_2OH^{+}, $FePC_4^{+}$
**20**	206.82	$Fe_2PO_4^{+}$, $Fe_2P_2O_2H^{+}$, $Fe_3C_2NH^{+}$, $Fe_3C_3H_3^{+}$
